# Influence of different dose calculation algorithms on the estimate of NTCP for lung complications

**DOI:** 10.1120/jacmp.v14i5.4316

**Published:** 2013-09-06

**Authors:** Emma Hedin, Anna Bäck

**Affiliations:** ^1^ Department of Radiation Physics Clinical Sciences University of Gothenburg Gothenburg Sweden; ^2^ Therapeutic Radiation Physics Medical and Biomedical Engineering Sahlgrenska University Hospital Gothenburg Sweden

**Keywords:** radiation therapy, pneumonitis, dose calculation algorithms, NTCP

## Abstract

Due to limitations and uncertainties in dose calculation algorithms, different algorithms can predict different dose distributions and dose‐volume histograms for the same treatment. This can be a problem when estimating the normal tissue complication probability (NTCP) for patient‐specific dose distributions. Published NTCP model parameters are often derived for a different dose calculation algorithm than the one used to calculate the actual dose distribution. The use of algorithm‐specific NTCP model parameters can prevent errors caused by differences in dose calculation algorithms. The objective of this work was to determine how to change the NTCP model parameters for lung complications derived for a simple correction‐based pencil beam dose calculation algorithm, in order to make them valid for three other common dose calculation algorithms. NTCP was calculated with the relative seriality (RS) and Lyman‐Kutcher‐Burman (LKB) models. The four dose calculation algorithms used were the pencil beam (PB) and collapsed cone (CC) algorithms employed by Oncentra, and the pencil beam convolution (PBC) and anisotropic analytical algorithm (AAA) employed by Eclipse. Original model parameters for lung complications were taken from four published studies on different grades of pneumonitis, and new algorithm‐specific NTCP model parameters were determined. The difference between original and new model parameters was presented in relation to the reported model parameter uncertainties. Three different types of treatments were considered in the study: tangential and locoregional breast cancer treatment and lung cancer treatment. Changing the algorithm without the derivation of new model parameters caused changes in the NTCP value of up to 10 percentage points for the cases studied. Furthermore, the error introduced could be of the same magnitude as the confidence intervals of the calculated NTCP values. The new NTCP model parameters were tabulated as the algorithm was varied from PB to PBC, AAA, or CC. Moving from the PB to the PBC algorithm did not require new model parameters; however, moving from PB to AAA or CC did require a change in the NTCP model parameters, with CC requiring the largest change. It was shown that the new model parameters for a given algorithm are different for the different treatment types.

PACS numbers: 87.53.‐j, 87.53.Kn, 87.55.‐x, 87.55.dh, 87.55.kd

## I. INTRODUCTION

Radiation therapy treatments are designed and optimized by considering both tumor control probabilities and normal tissue complications. Estimations of normal tissue complications can be conducted by calculating the normal tissue complication probability (NTCP) using a NTCP model. A NTCP model uses a mathematical expression to describe the relationship between the delivered physical dose and the biological effect in normal tissue. Model parameters are empirically derived by fitting the NTCP predictions for a specific endpoint to the observed clinical outcome for a population of treated patients. The patient population includes individual patient‐specific variations (such as radiation sensitivity) and therefore a large number of patients must be included to achieve good precision in the model parameter values. The accuracy of the NTCP estimates depends on the accuracy of the assessment of the delivered dose, as well as uncertainties related to the clinical data material (e.g., difficulties in diagnosis). A low prevalence of the endpoint studied also results in poor statistics.

Today, the most commonly used NTCP models correlate the properties of an organ dose‐volume histogram (DVH) to the probability of a radiation‐induced complication. The delivered organ dose is usually described by the DVH from the treatment planning system (TPS), and is thereby set equal to the planned dose. Due to limitations and uncertainties in the dose calculation algorithms, different algorithms can predict different dose distributions and DVHs for the same treatment. This can be a problem if published NTCP model parameters are used to estimate NTCP, but the dose calculation algorithm used at the clinic is different from that used to derive the parameters.

Several available published NTCP model parameters^1^, ^2^, ^3^ are based on dose calculations belonging to a simpler generation of correction‐based dose calculation algorithms, such as the pencil beam (PB) and the pencil beam convolution (PBC) algorithms in the Oncentra and Eclipse TPSs, respectively. These correction‐based pencil beam dose calculation algorithms have a more limited accuracy — especially in regions of a heterogeneous medium — compared to the collapsed cone (CC) and the analytical anisotropic algorithm (AAA) available in the same TPSs. The calculation of dose in lung tissue with different dose calculation algorithms are described well in the literature.^(^ ^4^ ^,^ ^5^ ^)^ For example, both PB and PBC have been shown to overestimate the dose in a low‐density material (e.g., the lung) compared to Monte Carlo calculations, while CC and AAA were shown to be in good agreement with Monte Carlo calculations in those regions.^(^ ^4^ ^,^ ^5^ ^)^

It has been concluded^6^, ^7^, ^8^ that different dose calculation algorithms yield different NTCP model parameter values, and that it is important to use NTCP model parameters that correspond to the selected dose calculation algorithm. It has also been reported^(7)^ that the difference in the NTCP model parameters can be comparable to the published uncertainties of the parameters. Brink et al.^(7)^ presented a method for deriving new algorithm‐specific NTCP model parameters; this method does not require information about treatment outcomes for a large number of treatments. Their approach is to determine which NTCP model parameters, in combination with the dose calculation algorithm of interest, result in the same NTCP values as those calculated with the original model parameters and original dose calculation. In particular, Brink and colleagues adjusted the NTCP model parameters from different studies on lung complications for an algorithm change from PB to CC. They compared their results, based on tangential breast treatment plans, to a study by De Jaeger et al.^(6)^ This latter study presented original NTCP model parameters for both a PB and a CC algorithm for a clinical data material for lung treatments. The similarities found in those comparisons were considered to support the validity of the method of refitting NTCP model parameters, as suggested by Brink et al.^(7)^ Such a conclusion assumes that the different lung dose distributions for the different types of treatments, tangential breast treatment and lung treatment, result in the same adjusted NTCP model parameters. This needs to be further validated.

NTCP model parameters for common TPS dose calculation algorithms other than CC and PB, such as AAA and PBC, are not available from the literature. The investigation of those four algorithms in one study would enable a comparison of these different generations of dose calculation algorithms. Furthermore, even though it is reasonable to assume that different implementations of simpler correction‐based pencil beam dose calculation algorithms (e.g., PB and PBC) would provide similar NTCP values, this has not yet been validated. The difference between algorithm‐specific model parameters needs to be examined in relation to the clinical uncertainties of the model parameters. An understanding of this relation will elucidate the importance of using model parameters derived for the same type of dose calculation algorithm as the one used to estimate NTCP.

The objective of this work is to determine how to change the NTCP model parameters for lung complications derived for a simple correction‐based pencil beam dose calculation algorithm, PB, in order to make them valid for CC, AAA, and PBC, using the method described by Brink et al.^(7)^ Model parameters for two NTCP models, relative seriality (RS)^(9)^ and Lyman‐Kutcher‐Burman (LKB),^(^ ^10^ ^,^ ^11^ ^)^ are collected from different published studies on different grades of pneumonitis.^1^, ^2^, ^3^, ^6^ The results for CC is compared to the results from Brink et al.^(7)^ and De Jaeger et al.^(6)^ Possible differences in the NTCP model parameters between PB and PBC are investigated. This work includes three types of treatments — tangential and locoregional breast treatment and lung treatment — to study how the results are affected by the type of treatment. The effect on NTCP of a different dose calculation algorithm is presented in relation to the reported uncertainties in the original model parameters.

## II. MATERIALS AND METHODS

Four different dose calculation algorithms are included in the study: (i) pencil beam convolution (PBC) with the inhomogeneity correction modified Batho, (ii) analytical anisotropic algorithm (AAA), (iii) pencil beam (PB), and (iv) collapsed cone (CC). The first two are in Eclipse v8.9 TPS (Varian Medical Systems, Palo Alto, CA), and the latter two are in Oncentra v4.0 TPS (Nucletron BV, Veenendaal, The Netherlands). A more detailed description and comparison of the algorithms can be found in Knöös et al.^(4)^ The calculation grid is 2.5 mm with a 5 mm slice thickness of the CT series. All algorithms are configured for the same Varian Clinac iX linear accelerator (Sahlgrenska University Hospital, Gothenburg, Sweden). The configurations are based on measured data acquired by an ionization chamber in water.

Three types of 3D conformal radiation treatment plans are analyzed: tangential breast (Tang), locoregional breast (LGL), and lung cancer (Lung). The Tang plans include two tangential 6 MV photon beams toward the breast. The LGL plans include additional six or 15 MV photon beams toward the axilla region (anterior and posterior beams). The treatment plans for the lung cases are individually optimized and vary from case to case. They are based on three beam directions — anterior, posterior, and from the ipsilateral side. All lung plans use a photon energy of 6 MV for all fields. The beam directions are optimized to restrict the dose to the spinal cord, the contralateral lung, and the heart. Additional beams from the contralateral side are added if needed.

**Figure 1:**
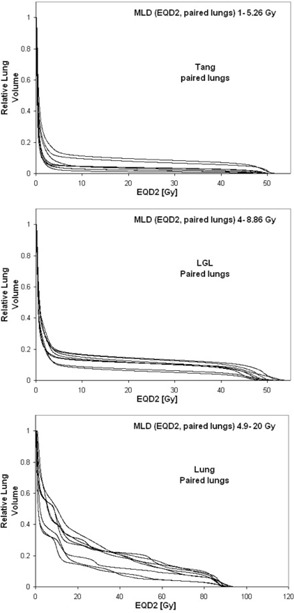
The ten DVHs (as calculated with PBC) for each of the three different treatment types: Tang, LGL, and Lung. The MLD range is given in each diagram.

**Figure 2:**
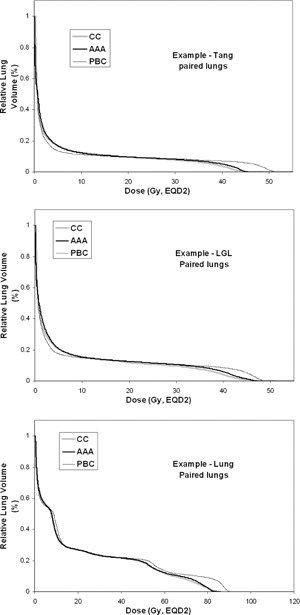
DVHs for PBC, AAA, and CC shown for one example of each treatment type (PB is omitted to facilitate viewing).

The study includes ten treatment plans of each type used for treatments at Sahlgrenska University Hospital. The plans are originally calculated with PBC. The plans are recalculated with AAA and also exported to Oncentra where they are recalculated with PB and CC. The monitor units obtained in the PBC calculation are used in all recalculations. Lung DVHs are compiled for each dose calculation algorithm in their respective TPS and used to estimate NTCP. The lung DVHs are corrected for fractionation effects according to the LQ model (α/β = 3, dose per fraction = 2 Gy). The LQ‐corrected doses are denoted as EQD2. GTV is subtracted from the lung DVH in the case of lung cancer. The DVHs are retrieved for paired lungs and, in the case of breast cancer treatment, additionally for the ipsilateral lung. The DVHs for the PBC calculated plans are shown in Fig. 1, and for comparison the DVHs for PBC, AAA, and CC are shown in Fig. 2 for one example of each treatment type (PB is omitted to facilitate viewing).

The mean lung dose (MLD), NTCP, and equivalent uniform dose (EUD)^(12)^ are calculated for all DVHs and for all four calculation algorithms. NTCP is calculated using the LKB‐model,^(^ ^10^ ^,^ ^11^ ^)^ with the DVH reduced to EUD, following Niemiero^(12)^ and the model parameters $\left[D_{50},\;m,\;n\right]$. NTCP is also calculated using the relative seriality (RS)^(9)^ model with the model parameters $\left[D_{50},\;\gamma ,\;s\right]$. Although NTCP adopts values between zero and unity, in this work they are presented as a percentage. The formulas used for the NTCP calculations are described in Eqs. (1) and (3) and the formula for calculating EUD for the NTCP model is described in Eq. (2), with notation following Rancati et al.^(13)^ $v_{i}$ is the fractional volume receiving the dose $D_{i}$.

(1) $$NTCP_{LKB}=\frac{1}{\sqrt{2\pi }}\int_{-\infty }^{t}e^{\frac{-x^{2}}{2}}dx$$

where $t=\frac{EUD-D_{50}}{mD_{50}}$ and

(2) $$EUD={\left(\sum_{i}v_{i}D_{i}^{1/n}\right)}^{n}$$

(3) $$NTCP_{RS}={\left[1-\prod_{i=1}^{M}{\left(1-P{\left(D_{i}\right)}^{s}\right)}^{v_{i}}\right]}^{1/s}$$

where *M* is the number of subvolumes (number of dose bins in the DVH), and $P(D_{i})=2^{-\text{exp}(e\gamma (1-D_{i}/D_{50})}$

The model parameters derived for a correction‐based pencil beam dose calculation algorithm are taken from four different publications describing studies that consider different grades of pneumonitis. Table 1 presents the model parameters used and a summary of the study characteristics, including the endpoint.

EUD and NTCP are calculated using the parameters from each published study. These parameters are applied on DVHs of the same treatment type (breast or lung cancer) and lung volume (paired lungs or ipsilateral lung), as used in the published studies. For the LKB model, a reduction of the DVH to EUD is performed as a step in calculating NTCP (see Eq. (2)). For the RS‐model, EUD is defined as the uniform dose that would yield the actual NTCP:

(4) $$EUD_{RS}=D_{50}*\frac{\left(1-\text{log}(-\text{log}(NTCP))\right)}{\text{log}(2)*e\gamma }$$

**Table 1:** Summary of the NTCP model parameter sets used in this study. Parameter values are found in Tables 2 and 3

		*Lung Volume*	${\text{MLD}}^{a}$ *Range(Gy)*	*Endpoint*	*Used on Treatment Type*
Seppenwoolde et al.^(1)^	LKB RS	paired paired	~ 2‐35	${\text{RP}}^{c}\;\leq$ grade 2 ${\text{SWOG}}^{d}\;$	Lung, LGL, Tang
Gagliardi et al.^(2)^	RS	ipsilateral	unknown	RP^c^ clinical	LGL, Tang
Rancati et al.^(3)^	LKB RS	ipsilateral ipsilateral	2.5–18	RP^c^ ≤ grade 1 modified CTC‐NCIC^e^	LGL, Tang
De Jaeger et al.^(6)b^	LKB	paired	~ 2–25	RP^c^ ≤ grade 2 SWOG^d^	Lung

a Paired lungs.

b Parameters for the octree/edge algorithm with equivalent path length inhomogeneity correction.

c Radiation pneumonitis.

d SouthWest Oncology Group toxicity criteria.

e Common Toxicity Criteria modified by the National Cancer Institute of Canada.

New NTCP model parameters for PBC, AAA, and CC were derived following the method suggested by Brink et al.^(7)^ The original parameters were assumed to be valid for PB.

All publications providing the NTCP parameters (Table 1), except for the one by De Jaeger et al.,^(6)^ present confidence intervals for the model parameters. The confidence interval for NTCP in this work is estimated by constructing a rectangular matrix of 1D maximum likelihood‐based confidence intervals for $D_{50}$ and m/y, and then searching for the maximum and minimum NTCP values yielded by any combination (the tissue‐describing parameter n/s is not considered). This is a simplified approach compared to the bundle method described by Gagliardi et al.^(2)^ and van Luijk et al.^(14)^ in which joint probability regions are used. Depending on whether the rectangle is encompassed by the joint probability region, the uncertainties could be slightly under or overestimated using a simplified approach where the 1D statistical uncertainties of the parameters are propagated through the NTCP functions.

The values of MLD and NTCP for the simple (PBC/PB) and sophisticated (AAA/CC) algorithms are compared for each TPS. The comparison is also made inter‐TPS using PB (in Oncentra) as a reference. The effect on the NTCP estimates of a different dose calculation algorithm is visualized by plotting the EUD shifts for a reference NTCP value. The effect is then related to uncertainties in NTCP by visualizing the confidence interval corresponding to the NTCP model parameters used. For the LKB model, it is straightforward to plot the NTCP values and corresponding confidence intervals against EUD. For the RS model, this operation is less natural, since a value of uncertainty in the NTCP calculated for a homogeneous irradiation does not necessarily hold for all DVHs of the same EUD (with the same NTCP).

## III. RESULTS

The estimated dose distribution and the corresponding DVH both change when the treatment plans are recalculated with a different dose calculation algorithm. A change from PBC to AAA causes an average relative decrease in MLD (1 SD) of 5% (± 2%), 4% (± 2%), and 4% (± 4%) for the Lung, LGL, and Tang plans, respectively. The corresponding results for a PB‐to‐CC change are 8% (± 2%), 9% (± 1%), and 10% (± 3%).

Without adjusting the model parameters, the estimated NTCP will in general become smaller when changing from a correction‐based pencil beam dose calculation algorithm (PB/PBC) to a more sophisticated algorithm (CC/AAA). However, the results are inconsistent for the Tang plans in Eclipse, as in this case a PBC‐to‐AAA change sometimes results in a higher NTCP value. This can be understood, since the lateral electron scatter is not properly taken into account in the PBC algorithm. PBC does not correctly predict the increased penumbra width in the lung; as a result, there is an overestimation of lung volume receiving high doses and an underestimation of lung volume receiving low doses.^(5)^ Consequently, AAA will compute a higher dose outside the field compared to PBC. In contrast, PBC computes a higher dose within the radiation field. In all investigated treatments, one part of the lung is in‐field and one part is outside the field. For the Tang beam geometry, some plans have a very limited amount of in‐field lung tissue which can lead to a higher AAA‐based NTCP value compared to the PBC‐based NTCP value.

A change in algorithm from simple (PBC/PB) to more sophisticated (AAA/CC) yields similar results in both Eclipse and Oncentra. The maximum absolute difference between NTCP values (without adjusting the model parameters) for the two types of algorithms is seen for LGL plans with a 6% (10%) difference for Eclipse (Oncentra). The absolute difference naturally increases for NTCP values closer to the steepest point of the NTCP curve. The LGL and Tang plans were evaluated for the complication of milder grade pneumonitis (i.e., the endpoint chosen by Rancati et al.^(3)^). These plans therefore yield the largest absolute differences. The relative difference (the difference in NTCP value divided by the NTCP value for the simpler algorithm) is similar over the investigated dose interval. The maximum relative difference is 28% (45%) for Eclipse (Oncentra). The two NTCP models used, LKB and RS, show similar results.

**Figure 3:**
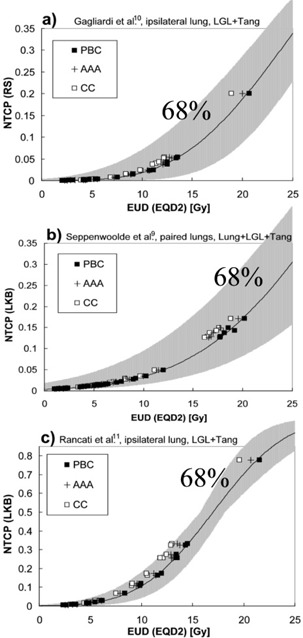
NTCP values plotted against EUD for different algorithms. The line shows the NTCP curve for the PB calculation and the model parameter set investigated in each respective diagram: (a) parameters from Gagliardi et al.^(2)^ (RS), ipsilateral lung, LGL+Tang plans; (b) parameters from Seppenwoolde et al.^(1)^ (LKB), paired lungs, Lung+LGL+Tang plans; (c) parameters from Rancati et al.^(3)^ (LKB), ipsilateral lung, LGL+Tang plans. Gray area represents the confidence interval with the level of confidence given in each diagram. Note: 3(c) has a y‐axis scale different from the others due to a much lower endpoint studied.

Examples of how the NTCP curves are shifted due to a change of dose calculation algorithm from PB (reference) to PBC, AAA, and CC are shown in Fig. 3. PB‐based NTCP values are plotted against the different values of EUD for the different dose calculation algorithms. Hence, the diagrams visualize the curve shift that is necessary to yield the same NTCP value for a PBC/AAA/CC‐calculated DVH as for the reference PB‐calculated DVH. Figure 3(b) includes all studied treatment plans. Figures 3(a) and 3(c) include only breast plans, since the NTCP model parameters were based on dose data for the ipsilateral lung in those cases. The differences in NTCP values between the figures are due to differences in the endpoint studied (notice the difference in y‐scales in Fig. 3). It is clear that the absolute differences in NTCP values at the lower end of the curve are very small. Seppenwoolde et al.^(1)^ and Rancati et al.^(3)^ give model parameters both for the RS and LKB models. The two models show analogous result; thus, only one model is shown in Fig. 3. The two pencil beam algorithms (PB and PBC) are similar, while AAA and CC show a larger change in NTCP value, with CC showing the largest change (see Fig. 3).

Figure 3 also presents the uncertainty of the original NTCP model parameters; the gray area symbolizes the confidence interval of the NTCP value for each EUD. In Figs. 3(a) and 3(b), the curve shifts for the different algorithms are relatively small compared to the confidence interval, while in Fig. 3(c) some shifts are comparable in size to the confidence interval. The reported confidence intervals of the NTCP model parameters differ between different studies. The smaller confidence interval in Fig. 3(c) can be due to the fact that studies on a mild and more frequent endpoint^(3)^ will have a high prevalence of the endpoint.

New algorithm‐specific NTCP model parameters have been derived. The PB algorithm is the reference algorithm in this study. The new parameters are shown in 2, 3 together with the corresponding original parameters. The differences observed between the parameters for PB and PBC are small (see Fig. 3). This expected result suggests that it may be applicable to use the same NTCP model parameters for NTCP estimates for both algorithms. AAA and CC require a change in the NTCP model parameters, with CC requiring the largest change (see Fig. 3 and 2, 3). The standard errors (68% confidence level) of the new parameters are small, which suggests that the chosen number of treatments per treatment type is sufficiently large (ten patients per treatment type). The change in the model parameters for a given change of dose calculation algorithm is observed to be dependent on the type of treatment. The new algorithm‐specific model parameters determined based on the original model parameters of Seppenwoolde et al.^(1)^ for the LKB model and of Gagliardi et al.^(2)^ for the RS model can be compared to the results presented by Brink et al.^(7)^ The Tang plan results in this study are similar to the Tang plan results presented by in the Brink study (within one standard error). The difference in the parameter $D_{50}$ presented for the algorithms in De Jaeger et al.^(6)^ is larger than the corresponding difference in $D_{50}$ for PB and CC determined in this study. This inconsistency could be due to the different design of lung treatment plans. Another cause could be the differences in methods used to derive the new model parameters in this study with the methods used in the De Jaeger study

**Table 2:** Refitted parameters for PBC, AAA, and CC with PB as a reference. Results for the LKB model

*LKB*		*Refitted for Treatment Type*	$D_{50}\pm 1\;\text{SE}$	m ± 1SE	*n*
Seppenwoolde et al^(1)^ (paired lungs) refitted for ${\text{CC}}^{a}$ (Brink et al^(7)^, PB ref)			30.80 26.80 ± 0.50	0.37 0.369 ± 0.001	0.99 0.99
	PBC	Lung	30.78 ± 0.19	0.370 ± 0.003	0.99
Paired lungs	AAA	Lung	29.19 ± 0.25	0.374 ± 0.004	0.99
	CC	Lung	28.40 ± 0.16	0.374 ± 0.003	0.99
	PBC	LGL	31.35 ± 0.17	0.371 ± 0.001	0.99
Paired lungs	AAA	LGL	29.40 ± 0.23	0.369 ± 0.001	0.99
	CC	LGL	27.92 ± 0.13	0.370 ± 0.000	0.99
	PBC	Tang	30.00 ± 0.13	0.370 ± 0.000	0.99
Paired lungs	AAA	Tang	28.33 ± 0.22	0.370 ± 0.000	0.99
	CC	Tang	27.00 ± 0.22	0.369 ± 0.000	0.99
Rancati et al^(3)^ (ipsilateral *lung*):			17	0.33	0.91
	PBC	LGL	17.10 ± 0.05	0.336 ± 0.004	0.91
Ipsilateral lung	AAA	LGL	16.40 ± 0.09	0.336 ± 0.006	0.91
	CC	LGL	15.50 ± 0.07	0.339 ± 0.005	0.91
	PBC	Tang	16.56 ± 0.08	0.332 ± 0.0009	0.91
Ipsilateral lung	AAA	Tang	15.47 ± 0.08	0.325 ± 0.0009	0.91
	CC	Tang	14.72 ± 0.08	0.325 ± 0.0011	0.91
De Jaeger et al^(6)^ (paired lungs) EPL^b^			34.10	0.45	1
De Jaeger et al^(6)^ (paired lungs) CS^c^			29.20	0.45	1
	PBC	Lung	34.08 ± 0.21	0.450 ± 0.003	1
Paired lungs	AAA	Lung	32.34 ± 0.28	0.454 ± 0.004	1
	CC	Lung	31.48 ± 0.17	0.454 ± 0.003	1

a Collapsed cone.

b Octree/edge algorithm with equivalent pathlength inhomogeneity correction in U‐MPlan.

c Convolution/Superposition algorithm in Pinnacle.

The standard errors presented refer to the mathematical uncertainties that stem from the refitting procedure.

**Table 3:** Refitted parameters for PBC, AAA, and CC with PB as a reference. Results for the RS model

*RS*		*Refitted for Treatment Type*	$D_{50}\;\pm \;1\;\text{SE}$	Y± 1SE	*s*
Seppenwoolde et al.^(1)^ (paired lungs):			34	0.9	0.06
	PBC	Lung	33.89 ± 0.20	0.902 ± 0.006	0.06
Paired lungs	AAA	Lung	32.02 ± 0.29	0.893 ± 0.009	0.06
	CC	Lung	31.21 ± 0.19	0.892 ± 0.006	0.06
	PBC	LGL	34.60 ± 0.17	0.897 ± 0.001	0.06
Paired lungs	AAA	LGL	32.45 ± 0.25	0.900 ± 0.002	0.06
	CC	LGL	30.74 ± 0.15	0.900 ± 0.001	0.06
	PBC	Tang	33.16 ± 0.15	0.899 ± 0.000	0.06
Paired lungs	AAA	Tang	31.00 ± 0.23	0.900 ± 0.001	0.06
	CC	Tang	29.58 ± 0.23	0.902 ± 0.001	0.06
Gagliardi et al.^(2)^ (ipsilateral lung)			30.1	0.966	0.012
refitted for ${\text{CC}}^{a}$ (Brink et al^(7)^, PB ref)			26.20 ± 0.40	0.972 ± 0.005	0.012
	PBC	LGL	30.49 ± 0.13	0.959 ± 0.003	0.012
Ipsilateral lung	AAA	LGL	29.23 ± 0.19	0.966 ± 0.004	0.012
	CC	LGL	27.57 ± 0.12	0.964 ± 0.003	0.012
	PBC	Tang	29.50 ± 0.15	0.962 ± 0.001	0.012
Ipsilateral lung	AAA	Tang	27.52 ± 0.17	0.974 ± 0.001	0.012
	CC	Tang	26.16 ± 0.18	0.973 ± 0.001	0.012
Rancati et al.^(3)^ (ipsilateral lung)			17.30	1.07	0.11
	PBC	LGL	17.34 ± 0.06	1.053 ± 0.016	0.11
Ipsilateral lung	AAA	LGL	16.55 ± 0.11	1.053 ± 0.027	0.11
	CC	LGL	15.62 ± 0.09	1.049 ± 0.022	0.11
	PBC	Tang	16.92 ± 0.09	1.058 ± 0.004	0.11
Ipsilateral lung	AAA	Tang	15.70 ± 0.08	1.092 ± 0.004	0.11
	CC	Tang	14.97 ± 0.09	1.092 ± 0.005	0.11

a Collapsed cone The standard errors presented refer to the mathematical uncertainties that stem from the refitting procedure.

## IV. DISCUSSION

The results in this study show that dose calculations with a correction‐based pencil beam algorithm will result in higher MLD and higher estimated NTCP for pneumonitis (without adjusting the model parameters) for the same treatment plan, compared to dose calculations with more sophisticated algorithms such as AAA and CC. Those results are consistent with other published results.^6^, ^7^, ^8^ For example, the reported average differences in MLD of 12% (± 2%) for tangential breast treatments^(7)^ and 16.6% (± 4.5%) for lung treatments^(6)^ can be compared to the average MLD differences for a PB‐to‐CC change of 8% (± 2%), 9% (± 1%), and 10% (± 3%) for this study's Tang, LGL, and Lung treatments, respectively. The effect on the absolute NTCP value of a different dose calculation algorithm (without adjusting the model parameters) is larger when the NTCP value is on the steeper part of the dose‐response curve (higher MLDs or lower endpoint). For treatment plans with small MLDs (e.g., Tang plans), the effect on the absolute values of NTCP is so small that it can be considered clinically irrelevant.

The change in NTCP model parameters when changing from PB to AAA or CC is most often smaller than the reported confidence interval of the model parameters, but could in some cases be similar in size. This has been discussed earlier,^(7)^ and the effect is clearly seen in this study. The reported confidence intervals of the NTCP model parameters differ between studies. For example, studies on a mild and more frequent endpoint^(3)^ will have high prevalence of the endpoint; this could result in small confidence intervals for the model parameters. The actual accuracy of the models and the model parameters from the published studies and their ability to reproduce correct values of NTCP are not investigated here, as they are beyond the scope of this study. The new algorithm‐specific model parameters presented in this study will not result in more accurate NTCP estimates than the original parameters from the published studies; they can only prevent the introduction of an additional uncertainty due to differences in the dose calculation algorithms. The uncertainty in the model parameter values provides information regarding the precision of the NTCP estimate that can be obtained using those parameters. The introduction of additional uncertainties should be avoided, even if they are small compared to the uncertainties in the model parameter values. Since the additional error due to differences in dose calculations can be of comparable magnitude to the uncertainties in the model parameter values, it is important to use model parameters derived for the same type of dose calculation algorithm as the one used to estimate NTCP.

Improvements in the dose calculations is a necessary step towards finding the true dose‐response relationship, but it does not improve the estimation of NTCP without reconsidering how the biological effect is modeled or retrieving new parameters to already existing models. Furthermore, the delivered dose to the lung is usually described by the DVH from the TPS, and is thereby estimated to the planned dose rather than the actual delivered dose. Uncertainties in the assessment of the delivered dose include the uncertainties correlated to the accuracy of the dose calculation, as well as uncertainties involved in the dose delivery (e.g., patient positioning and internal organ motion). For the lung, there is also a periodic change in organ density due to respiration, which tends not to be considered. All uncertainties in the assessment of the delivered dose should be taken into account to further improve the estimate of NTCP and to determine the true dose‐response relationship.

The shape of the lung DVH can vary for different types of treatments. For example, the DVH for a Tang treatment plan has a different shape than that for a Lung treatment plan (Fig. 1). The DVHs for different Tang treatment plans vary somewhat in dose level, but they have similar shapes. The same trend holds for the LGL treatment plans; however, the DVHs for different Lung treatment plans vary in both dose level and shape. This is a reflection of the fact that the design of the treatment plans in terms of beam direction is similar for all Tang treatment plans and LGL plans, respectively, while it is more individual for the Lung treatment plans and depends on the size and location of the tumor. The influence of the shape of the DVH on the NTCP estimates is small due to the design of the NTCP models, especially for the LKB model in which the DVH is reduced to one representative dose value. An analysis that considers the different ways of taking the DVH shape into account in the LKB and RS models is beyond the scope of this study.

However, it is important here to discuss how the DVH will change when moving from a correction‐based pencil beam type of dose calculation (for example, PB or PBC) to a more sophisticated algorithm (AAA or CC). The change in DVH will depend on the design of the treatment plan and the photon energy used. PB and PBC have more limited accuracy for modeling radiation transport, and those limitations will be more or less pronounced in the lung dose distribution for different treatment plans and patient geometries. For example, PB and PBC erroneously predict a higher dose in the lung region close to the border between the lung and higher density tissues.^(5)^ The fraction size of the irradiated lung volume that is close to the higher density tissues will influence the effect on the DVH shape due to a different dose calculation algorithm. Another example is that PB and PBC do not correctly predict the increased penumbra width in the lung; this causes an overestimate of lung volume receiving a high dose and an underestimate of lung volume receiving a low dose.^(5)^ Consequently, the fraction size of the penumbra regions in the lung influences the effect on the DVH shape. The change in DVH due to a different dose calculation algorithm can therefore be dependent on the type of treatment. For example, even if the DVHs for two different treatment types have the same shape for one dose calculation algorithm, they will not necessarily have the same shape if they are recalculated with another dose calculation algorithm. Therefore, the effect on NTCP due to a different dose calculation algorithm can also be dependent on treatment type.

Furthermore, the change in DVH can be different for different Lung plans, but will most likely be similar for all Tang and LGL treatment plans. The PB‐to‐CC change in the model parameters from Gagliardi et al.^(2)^ was reported by Brink et al.^(7)^ based on tangential breast cancer treatments. The result noted in the study by Brink and colleagues is consistent within one standard error with the results reported for the Tang plans in this study. The differences found in the new model parameters for the LGL and the Tang plans reported in this work could be due to the differences in the treatment types. The PB‐to‐CC change in model parameters reported in this study for Lung treatment plans differ by more than one standard error from the corresponding results reported by De Jaeger et al.^(6)^ Differences in results between this study and the De Jaeger study can be due to differences in the design of lung treatment plans and/or limitations in the method used to derive new model parameters.^(7)^ It is not possible to distinguish which reason is the main cause for the differences. In practice, another possible cause could be a difference in the implementation of the dose calculation algorithms.

## V. CONCLUSIONS

The error that can be introduced in NTCP estimates due to differences in dose calculation algorithms can be of the same magnitude as the confidence intervals of calculated NTCP values. The use of algorithm‐specific NTCP model parameters can prevent the introduction of this additional uncertainty. The change in NTCP model parameters for lung complications when changing from PB (Oncentra TPS) to PBC (Eclipse TPS), AAA (Eclipse TPS), or CC (Oncentra TPS) are presented for three different treatment types. The same NTCP model parameters can be used for both PB and PBC with good accuracy.

## ACKNOWLEDGMENTS

This work is supported with funding from the King Gustaf V Jubilée Clinic Cancer Research Foundation, the Percy Falk Foundation, and the Assar Gabrielssons Foundation, which are gratefully acknowledged. The authors are also thankful for the support from and fruitful discussions with Roumiana Chakarova, medical physicist at Sahlgrenska University Hospital, Gothenburg.
